# PrediQt-Cx: Post Treatment Health Related Quality of Life Prediction Model for Cervical Cancer Patients

**DOI:** 10.1371/journal.pone.0089851

**Published:** 2014-02-26

**Authors:** Satwant Kumar, Madhu Lata Rana, Khushboo Verma, Narayanjeet Singh, Anil Kumar Sharma, Arun Kumar Maria, Gobind Singh Dhaliwal, Harkiran Kaur Khaira, Sunil Saini

**Affiliations:** 1 Institute of Bioinformatics, Bangalore, Karnataka, India; 2 Adesh Institute of Medical Sciences and Research, Bathinda, Punjab, India; 3 Himalayan Institute of Medical Sciences(HIMS), Dehradun, Uttarkhand, India; 4 Rabindra Nath Tagore Medical College, Udaipur, Rajasthan, India; Banaras Hindu University, India

## Abstract

**Background:**

Cervical cancer is the third largest cause of cancer mortality in India. The objectives of the study were to compare the pre and the post treatment quality of life in cervical cancer patients and to develop a prediction model to provide an insight into the possibilities in the treatment modules.

**Methodology/Principal Findings:**

A total of 198 patients were assessed with two structured questionnaires of Health Related Quality of Life (The European Organisation for Research and Treatment of Cancer, EORTC QLQ C-30 and CX-24). The baseline observations were recorded when the patients first reported (T1) and second evaluation was done at 6 months post treatment (T2). The mean age of detection was 50.9 years with the literacy level being non-educated or less than high school. Majority of them were married/cohabiting 179 (90.4%). On histopathological examination (HPE) squamous cell carcinoma was found to be the most common cell type carcinoma 147 (74.2%) followed by Adenocarcinoma 31 (15.7%). Radical hysterectomy was the most common treatment modality 76 (38.4%), followed by Wertheims Hysterectomy 46 (23.2%) and Radiochemotherapy 59 (29.8%). The mean score of global health of cervical cancer patients post treatment was 77.90, which was significantly higher than the pre - treatment score (54.32). Mean “symptoms score” post treatment was 21.69 with an aggravation of 7.32 compared to pre treatment scores. Patients experienced substantial decrease in sexual activity post treatment.

**Conclusions/Significance:**

The prediction model(PrediQt-Cx), based on Support Vector Machine(SVM) for predicting post treatment HRQoL in cervical cancer patients was developed and internally cross validated. After external validation PrediQt-Cx can be easily employed to support decision making by clinicians and patients from north India region, through openly made available for access at http://prediqt.org.

## Introduction

### Background

Cervical cancer is one of the most common cancers worldwide [1]. The disparity between the mortality rates amongst the high and the low income group countries signifies a health inequity [1]. Further, there exists a higher prevalence in the low socio-economic groups within different countries, which exemplifies uneven availability and accessibility to the health services [2].

### Situation

The age-adjusted incidence of cervical cancer in India is 25.9% (1, 34,420 incident cases), which is higher than the average for South East Asian region [3]. According to the Population Based Cancer Registries (PBCR), it has the second highest prevalence following breast cancer [4]. The pervasiveness of cervical cancer is much higher in rural and low socioeconomic groups in India [2], [5]. Lack of access to screening, affordability, compliance and follow up of treatment are the major causes for this pattern of discordance [6].

### Economic Burden in India

In addition to the significant contribution to the mortality rates, cervical cancer also leads to the loss of productive life due to prolonged disability [1]. It contributes to considerable economic burden, as the women of age group 25 - 64 years tend to be the sole caretakers of their families and households; and in some cases, they as well contribute to the family income [7]. Additionally, they are further deprived due to high medical costs, especially since most of the cases in developing countries are diagnosed at later stages,when the treatment is costly combined with poor prognosis [8]. Accordingly, there is a need to prevent the soaring secondary costs and economic burden as the more cost effective treatment modality can effect the outcome of patient’s post treatment quality of life.

The current study compares the scores of pre-treatment and post-treatment quality of life. The quality of life has been documented to vary with the treatment, the time since diagnosis and the cancer site [9]. Additionally, there are cultural differences which affect the quality of life assessment perception, as there are dissimilar beliefs regarding the constituents of normality and disease [10]. Being diagnosed with the disease is associated with social stigma, due to the belief that it is caused by sexual promiscuity, poor hygiene and the use of oral contraceptives [11]. Given that these beliefs are different from the Western world, therefore, reasonably there should be differences in the perception of quality of life in India. Thus, we conducted the study in patient population of north India and determined whether there is any significant difference in the post treatment quality of life. Furthermore, we structured a prediction model based on the findings of our study, which would serve to forecast the quality of life.

## Materials and Methods

### Ethics Statement

Institutional approval was obtained after reviewing the study protocol by the Institutional Review Board of the Himalayan Institute of Medical Sciences and Research, Uttarkhand. Written informed consent were obtained from patients after clear and concise explanation about the study. All the women in this study were informed about the right to refrain from participation in the study without any affect on the quality of health care services being provided to them. Patient confidentiality was assured by coding the patients’ information and removing the identifiable personal data before the data analysis.

### Study Population

All patients who had undergone surgical treatment for cervical cancer between April, 2007 to March, 2011 at the tertiary care academic hospital, Himalayan Institute of Medical Sciences and Research, Uttarkhand in Northern India were selected for this study. Of the 361 patients who gave completely filled the consent form, 108 were excluded due to non-availability of post treatment assessment data. 55 subjects were excluded due to accompanying psychiatric disease, cognitive impairment, diagnosis of multiple cancers, ambiguity in determining follow-up time or incomplete information, and Stage IV of cancer. After exclusion, 198 subjects with cervical cancer were included in the study group.

### Measures and Instruments

The patients were interviewed using two structured questionnaires-Health Related Quality of Life (HRQoL) questionnaires (EORTC QLQ C-30 and CX-24), to collect information about socio-economic and clinical status. The baseline values were recorded when the patient first reported to the hospital (T1) and second evaluation was done at a follow-up visit 6 months post treatment (T2). Clinical evaluation was performed at the same time points and relevant information was documented.

The European Organisation for Research and Treatment of Cancer (EORTC)- general cancer quality of life score questionnaire (QLQ C-30, and its cervical cancer module (QLQ CX-24) were used to measure HRQoL. These questionnaires have been extensively tested in multicultural and multidisciplinary settings, and have been confirmed to be reliable and valid [12]–[14]. The EORTC QLQ C-30 questionnaire comprises of 30 questions which assess functioning (physical, role, cognitive, emotional, social) and symptoms (fatigue, nausea and vomiting, pain, dyspnea, insomnia, appetite loss, constipation, diarrhea, financial difficulty), and a global health status score which assesses the overall QOL. The EORTC QLQ CX-24 questionnaire consists of 24 questions assessing functioning (body image, sexual enjoyment, sexual/vaginal functioning) and symptoms (symptoms experience, lymphoedema, peripheral neuropathy, menopausal symptoms, sexual worry). Both questionnaires use a four-point response scale (not at all, a little, quite a bit, and very much) to assess each functional or symptom item, and a seven-point response scale is used to assess global health status (from very poor to excellent). For model development the categorical raw scores were linearly transformed into a score of 0 - 100 for processing according to the EORTC manual [15].

Systemic literature review, established clinical knowledge and by performing univariate linear regression analysis following features were found to be statistically significant 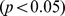 and were used in the development of the prediction model: age, marital status, vaginal bleeding, vaginal discharge, dyspareunia, abdominal pain, weight loss, parity, difficulty in controlling bowels or emptying bladder, cervical cancer stage, treatment, lymphoedema and peripheral neuropathy.

### Prediction Model Development

For the development of the prediction model we compared the performance of 4 different algorithms. Support Vector Machines(SVM) was utilized with two different kernels- Linear and Radial Basis Function(RBF), Artificial Neural Network(ANN) and regularized Logistic Regression(LR). The preprocessing of the dataset was performed to standardize and normalize the features before applying learning algorithms. The quality of life questionnaire mean results were obtained in continuous data format on a scale of 0 - 100, which was converted into discrete 3 class output for each scale, to train the classifier machine learning algorithms (See Appendix S1). All the 13 variables were provided to the models and trained separately for functional, symptom and global health/QoL scales. The Scikits-Learn module in Python programming language was utilized for the development of Logistic Regression and SVM models [16]. The ANN multilayer perceptron(MLP) was developed using PyBrain module in Python [17].

### Prediction Model Validation and Comparison

Cross-validation method with stratified K-Fold iterator was employed to derive a reliable estimate of the performance. Cross-validation approach splits the whole data several consecutive times in different train and test set, and returns the averaged value of the prediction scores. Stratified K-Fold return group of samples, called “folds” by preserving the same percentage for each target class as in the complete set. The stratified 6-folds were implemented to estimate the accuracy. Predicted values were then combined across the 6 runs and summarised by mean Area Under Curve(AUC). The mean accuracy, mean area under the curve (AUC),mean squared error (MSE) and adjusted-for-chance mutual information index (AMI) of each of the models were calculated for performance comparison between the Logistic Regression, SVM (‘Linear’), SVM (‘RBF’) and ANN models. An individual cervical cancer patient was the unit of analysis in this study. The area under the ROC (Receiver Operating Characteristics) curve or simply AUC has been validated to evaluate the ranking performance of the machine learning algorithms [18]. The MSE, which is computed as difference between the true and predicted values and then averaged across data, was used as an indicator of effectiveness with model fits [19]. Adjusted Mutual Information (AMI) is an adjustment of the Mutual Information (MI) score to account for chance [20](See Appendix S1).

### Statistical Analysis

We used R, an open source statistical programming environment for univariate linear regression analysis; and scientific computing Python packages to generate logistic regression, SVM and ANN models. [16], [17], [21], [22]. The student’s 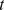 test or 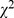 test was utilized for comparing the characteristics in the study population and the quality of life scales before and after treatment 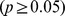.

## Results

The clinical and demographic characteristics of the participant cervical cancer patients are listed in Table 1. The quality of life of cervical cancer patients in the functioning scales comprising physical, emotional and social functioning were statistically significant post treatment. Furthermore, the global health/QoL improvement was statistically significant, while cognitive functioning was not substantial as presented in Table 2. The mean score of global health of cervical cancer patients post treatment was 77.90, which was significantly higher than pre treatment values 54.32 by about 23.58 points. The symptom scales items- “fatigue” and “nausea/vomiting” improved but the symptom “pain” was aggravated. Post treatment single item scales items- “dyspnoea”, “insomnia”, “appetite loss” and “constipation” were lower, while the item - financial difficulties was elevated in comparison to pre treatment scores. Table 3 represents cervical cancer specific EORTC QLQ CX-24 module scores for cervical cancer patients pre and post treatment. The patient experienced enhanced body image, however sexual functions like sexual activity and sexual/vaginal functioning were decreased. Symptoms experience- “lymphedema”, “peripheral neuropathy”, “menopausal symptoms” and “sexual worry” of the patients aggravated variably post treatment. Mean symptoms experience score post-treatment was 21.69, with an aggravation of 7.32 compared to pre-treatment scores. The patients experienced substantial decrease in sexual activity functioning with post-treatment mean score of 11.52 compared to 24.17 pre-treatment.

**Table 1:** Demographic and clinical characteristics of the patient cohort*.

Variables		N(%)
Age (years)		50.9±10.4
Education	No education	74(37.4)
	Less than High School	77(38.9)
	High school and above	47(23.8)
Marital Status	Married/Cohabiting	179(90.4)
	Not Married	19(9.6)
Menopausal Status	Premenopausal	63(31.8)
	Postmenopausal	135(68.2)
Parity	Nullipara	32(16.7)
	Primipara	92(46.5)
	Multipara	74(36.8)
Abdominal Mass	No	172(86.7)
	Yes	26(13.1)
Staging(FIGO)†	Stage IA1	3(1.5)
	Stage IA2	11(5.6)
	Stage IB1	53(26.8)
	Stage IB2	21(10.6)
	Stage IIA1	26(13.1)
	Stage IIA2	21(10.6)
	Stage IIB	27(13.6)
	Stage IIIA	13(6.5)
	Stage IIIB	26(13.1)
Cell Type	Squamous cell carcinoma	147(74.2)
	Adenocarcinoma	31(15.7)
	Adenosquamous cell carcinoma	6(3.0)
	Other types	14(7.1)
Treatment Modality‡	Conization	3(1.5)
	Total Abdominal Hysterectomy	8(4.0)
	Radical Hysterectomy	76(38.4)
	Chemotherapy	9(4.6)
	Wertheim’s Hysterectomy	46(23.2)
	Radiochemotherapy	59(29.8)

*Values are means ± standard deviations.

† FIGO, International Federation of Gynecology and Obstetrics.

‡ Some patients received multiple treatment interventions.

**Table 2:** Comparative pre and post treatment EORTC QLQ C-30 Quality of Life scores in cervical cancer women.

EORTC QLQ C-30Scale	PreTreatment	PostTreatment	P-Value*
**Global Health** **Status/Qol scale**			
Global Health Status/Qol	54.32(9.65)	77.90(7.17)	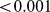
**Functional Scale**			
Physical functioning	69.86(10.73)	80.60(27.06)	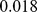
Role Functioning	69.06(16.68)	80.37(15.16)	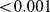
Emotional Functioning	60.20(16.37)	77.13(14.15)	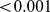
Cognitive Functioning	71.06(16.68)	73.39(18.36)	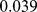
Social Functioning	60.67(11.57)	68.26(16.78)	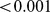
**Symptom scales/Items**			
Fatigue	38.21(11.15)	24.88(7.32)	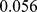
Nausea and Vomiting	12.01(11.30)	2.00(5.52)	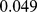
Pain	19.67(18.32)	22.66(6.23)	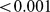
**Single Item scales**			
Dyspnoea	26.52(21.51)	10.56(15.86)	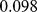
Insomnia	29.67(18.84)	22.30(24.12)	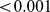
Appetite loss	33.98(17.67)	31.20(8.36)	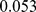
Constipation	33.43(20.60)	25.87(10.04)	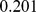
Diarrhoea1	3.87(9.67)	8.67(12.25)	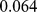
Financial Difficulties	34.67(28.62)	46.34(19.71)	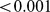

*P values are comparisons between groups with 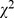 or student’s 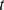 test.

Higher scores in the functioning and global health status scales represented better functioning and QOL, whereas higher scores in the symptom scales indicated greater problems.

**Table 3:** EORTC QLQ CX-24 cervical cancer module scores in cervical cancer woman pre and post treatment.

EORTC QLQ CX-24Scale	PreTreatment	PostTreatment	P-Value*
**Functional Scale**			
Body Image	18.36(15.32)	27.81(08.43)	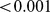
Sexual activity	18.54(18.67)	12.27(10.67)	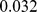
Sexual enjoyment	29.56(12.89)	10.33(06.81)	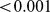
Sexual/vaginal functioning	24.42(8.98)	11.98(7.76)	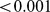
**Symptoms Scale**			
Symptoms experience	15.44(12.23)	28.65(17.42)	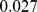
Lymphoedema	1.33(6.54)	13.87(19.24)	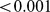
Peripheral neuropathy	4.33(6.47)	13.33(19.24)	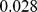
Menopausal symptoms	9.33(15.27)	34.67(22.52)	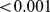
Sexual worry	14.66(16.88)	33.43(25.46)	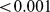

*P values are comparisons between groups with 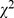 or student’s 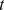 test.

Higher scores in the functioning and global health status scales represented better functioning and QOL, whereas higher scores in the symptom scales indicated greater problems.

### Predictive Factors Significance

The significance of the predictive factors previously selected using univariate linear regression analysis were compared. Table 4 shows the coefficients of significant variables for Symptom scale, Functional scale and Global health/Quality of Life (GH/QoL) post treatment in the logistic regression model. All the selected variables were found to be statistically significant (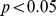).

**Table 4:** Coefficients(coef) of significant variables for Symptom scale, Functional scale and Global health/Quality of Life (GH/QoL) post treatment in Logistic Regression model, a Generalized linear model (GLM) type.

	Symptom Scale		Functional Scale		Global Health Scale	
Variables	Coef	P value	Coef	P value	Coef	P value
Age	1.1024	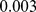	−0.2036	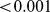	−1.1019	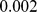
Marital Status	0.6136	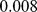	1.1539	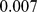	0.2683	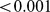
Vaginal Bleeding	2.1313	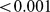	1.6091	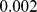	3.0775	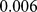
Vaginal Discharge	1.1313	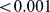	0.4091	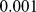	1.0876	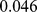
Dyspareunia	0.1223	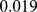	0.4415	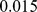	1.0322	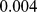
Abdominal Pain	2.3022	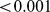	−1.1075	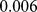	−2.1603	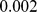
Weight Loss	0.1813	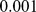	−0.2069	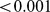	−2.2733	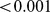
Parity	−0.1479	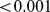	−1.0481	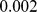	−1.0221	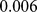
Bowel and Bladder control	3.0422	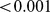	−2.075	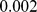	−1.0603	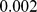
FIGO staging	3.2438	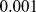	3.3078	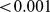	4.0627	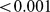
Treatment given	1.3407	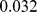	−4.0788	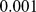	−3.3527	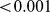
Lymphoedema	2.1813	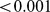	−1.2069	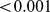	−0.27733	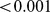
Peripheral Neuropathy	1.2561	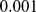	−1.3315	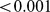	−0.3511	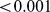

### Comparison of Prediction Models

Table 1 shows the performance comparison of four machine learning algorithms on Symptom, Global health/QoL and Functional scales for the prediction of post-treatment cervical cancer quality of life outcomes. This has been demonstrated in terms of Mean Squared Error (MSE), mean Area Under Curve (AUC), Adjusted for chance Mutual Information index (AMI) and accuracy for prediction.

#### Symptom scale

**Figure 1:**
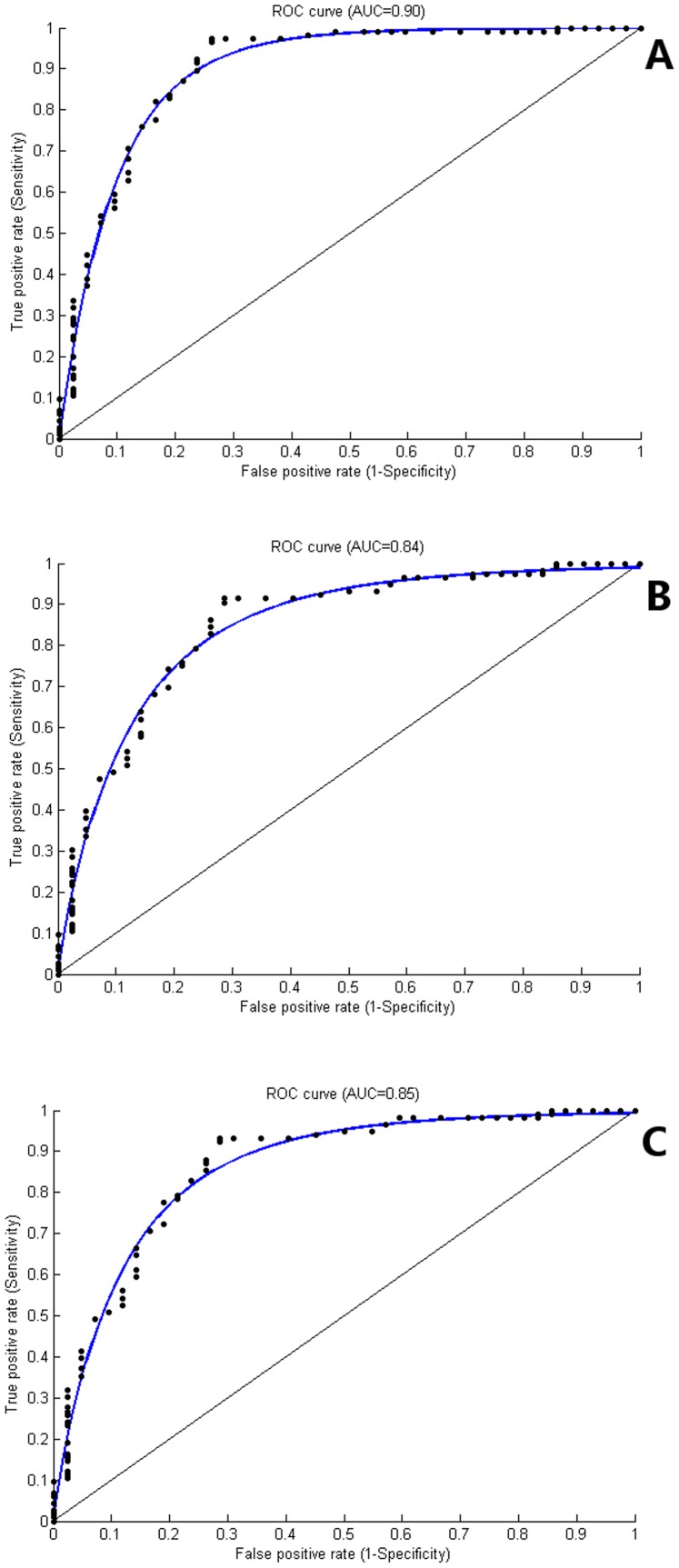
Mean Area Under Receiver Operating Characteristic (ROC) curve. Mean ROC (AUC) for Support Vector Machine algorithm with Linear Kernel for (A) Prediction of Symptom scale was 0.90. (B) Prediction of Global Health/QoL was 0.84. (C) Prediction of Functional scale was 0.85.

Comparison of the four models reveal, that all the models performed suitably on the Symptom scale. On the basis of accuracy, 3 models Support Vector Machine with Linear kernel-SVM (Linear), Support Vector Machine with Radial Basis Function-SVM (RBF) and LR (Logistic Regression) scored 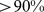; and SVM (Linear) outperformed other models with accuracy of 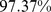. In terms of mean AUC, SVM (Linear) and Artificial Neural Network (ANN) surpassed SVM (RBF) and LR (mean AUC = 0.90, 0.85, 0.80 and 0.72, respectively). The mean squared error (MSE) was low for all four models varying between 0.02
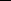
0.03. SVM (Linear) was superior to other models SVM (RBF), LR and ANN in terms of AMI (AMI = 0.90, 0.81, 0.82 and 0.82, respectively). (Table 5, Figure 1A).

**Table 5:** The performance comparison of four machine learning algorithms on symptom, global health/QoL and functional scales for the prediction of post treatment cervical cancer quality of life outcomes.

Scale	MSE	Mean AUC	AMI	Accuracy %
**Symptom Scale**				
SVM(Linear)	0.02	0.90	0.92	97.37
SVM(RBF)	0.03	0.80	0.81	94.58
LR	0.02	0.72	0.82	94.34
ANN	0.03	0.85	0.82	87.56
**Global Health/QoL**				
SVM(Linear)	0.07	0.84	0.79	95.26
SVM(RBF)	0.08	0.80	0.20	93.12
LR	0.13	0.64	0.59	89.29
ANN	0.08	0.73	0.65	74.38
**Functional Scale**				
SVM(Linear)	0.13	0.85	0.77	95.81
SVM(RBF)	0.26	0.90	0.78	97.32
LR	0.16	0.60	0.34	93.12
ANN	0.13	0.83	0.90	71.28

MSE = Mean Squared Error, AUC = Mean Area Under ROC(Receiver Operating Characteristics) Curve, AMI = Adjusted-for-chance Mutual Information Index, SVM(Linear) = Support Vector Machine with Linear Kernel, SVM(RBF) = Support Vector Machine with Radial Basis Function Kernel, LR = Logistic Regression, ANN = Artificial Neural Network.

#### Global Health/QoL scale

On Global Health/QoL scale SVM(Linear) was better than the other models in all the four parameters (MSE, Mean AUC, AMI and Accuracy equal to 0.07, 0.84, 0.79 and 95.26%, respectively). While SVM (RBF) was better than LR and ANN on all parameters except AMI (MSE, Mean AUC, AMI and Accuracy% = 0.08, 0.80, 0.20 and 93.12, respectively). In case of LR mean AUC (0.64) suffered, while ANN performed average (MSE, Mean AUC, AMI and Accuracy% = 0.08, 0.73, 0.65 and 74.38, respectively). On Global Health/QoL scale, performance of all the models suffered compared to other scales. (Table 5, Figure 1B).

#### Functional scale

The Functional scale SVM (RBF) did better than others on all parameters except MSE (MSE, Mean AUC, AMI and Accuracy% = 0.26, 0.90, 0.78 and 97.32, respectively). While SVM(Linear) sustained it’s performance on Functional scale as well (MSE, Mean AUC, AMI and Accuracy% = 0.13, 0.85, 0.77 and 95.81, respectively). ANN was better than LR except on Accuracy% (MSE, Mean AUC, AMI and Accuracy% = 0.13, 0.83, 0.90 and 71.28 and 0.16, 0.60, 0.34 and 93.12, respectively) (Table 5, Figure 1C).

As SVM (Linear) performed consistently on all the 3 functional scales, we utilized it in developing prediction model.

## Discussion

Our study compares the pre-treatment and the post-treatment HRQoL for cervical cancer patients in north India. Various factors are responsible for the changes in the quality of life of the women diagnosed with gynaecological cancer. During pelvic surgery, there is functional damage and removal of parts of the female genital tract. Additionally, radiation damages the vaginal mucosa and epithelium. Moreover, there are other side effects of radiotherapy such as nausea, vomiting, diarrhoea, constipation, mucositis, weight changes and hormonal changes [23].

For patients with disease of limited volume, radical abdominal hysterectomy is preferred, which impairs the quality of life due to physiological and psychological effects [24]. In the current study, it was found that the most common treatment modality opted was radical hysterectomy followed by, Wertheims hysterectomy and radio-chemotherapy. Almost 25% of the patients with early stage cancer reported post surgery changes in vagina that lasted for 5 years [24]. The concomitant radio-chemotherapy is responsible for doubling the acute and late toxicities [25].

The psychological impact is an essential parameter, as it determines the self perceived changes in the quality of life which are assessed. There was a preponderance of married or cohabiting women, while the non-married women constituted a small proportion of the affected population. Although, it may be held responsible for being a risk factor however, marital status has been found to be a predictive factor in “concerns” domain, which demonstrates that the presence of a partner provides emotional support for the patients [26]. It is suggested that the health professionals should give more importance to the role of family and spouse while quantifying the quality of life [26].

The Global Health Status showed a highly significant increase after the treatment making it obvious that quality of life improves after the treatment. Among the Functional Scales all the items confirmed a significant increase within 6 months, namely physical, role, emotional, cognitive and social functioning. This was in contrast to a study which revealed that the Global QOL, emotional and role functioning remained low even after 1 year of the completion of the treatment [27]. Nevertheless, role and social functioning illustrated a highly significant rise post treatment. This implies that the survivors perceived an enhancement in their public and civil roles and find a definitive improvement post-treatment in this regard.

The Symptom scale analysis revealed that there was a significant decrease in “fatigue” and “nausea/vomiting”. However, there was a highly significant rise in post-treatment “pain”. Amongst the items of Single Item Scale- “Appetite loss” decreased significantly. In contrast, in an another study using EORTC QLQ C-30 [28], the “level of nausea/vomiting”, “pain” and “appetite loss” were increased. In addition, there was an increase in post-treatment diarrhoea, however constipation decreased. Radiotherapy has been documented to be associated with diarrhoea while constipation is attributed to injury to the parasympathetic nerves during pelvic surgery [28], [29]. These findings may be affected by the relative number of patients receiving different treatment modalities and the individual differences in response to the therapies. A highly significant reduction in insomnia was depicted post-treatment. This may imply a reduction in the anxiety regarding the course of cancer. Conversely, the “financial difficulties” increased in a highly significant proportion of the survivors. Cervical cancer patients have shown to have significant difficulties with the finances [30]. This bears a specific relevance in developing countries, where the economic burden is a significant factor affecting the quality of life. Our study revealed that the survivors had a worse “body image” as compared to pre-treatment, this probably is a consequence of the cancer experience or the treatment as reported in the EORTC QLQ CX-24 scale.

Sexuality is an important aspect of gynaecological cancer, thus being a crucial determinant of the quality of life. The survivors have intimacy issues and are unacquainted so as how to recreate the intimate side of the relationship with their spouse. They have a constant fear of recurrence, combined with the grief of never having a child once diagnosed [31]. There was a significant decrease in “sexual activity”, along with a highly significant decrement in “sexual and vaginal functioning” score in our study. This is supported by the previous study which stated that approximately 40% to 100% individuals face sexual dysfunction after diagnosis and treatment. The reason behind the same is, that cervical cancer and its treatment affects the same areas of the body which are involved with sexual response [30]. It is well documented that both chemotherapy and radiotherapy are associated with sexual problems like dyspareunia, anxiety about sexual performance and insufficient lubrication [30]. Additionally, chemotherapy side effects, like nausea and fatigue may also reduce sexual functioning [32]. Various studies reinforce that the women who undergo surgery and receive radiotherapy as well have the worse sexual problems, as opposed to the women who are treated by surgery alone [33], [34]. This signifies that the patient, regardless of their treatment modality, should be counselled prior and post-treatment. Moreover, the treatment modality selection is of primary essence, so as to avoid the need of adjuvant radiotherapy after surgery.

The survivors also reported a highly significant rise in the “menopausal symptoms”. These menopausal symptoms were worse for the women with radiation therapy, as supported by a previous study stating more aggressive menopausal symptoms when patients are rendered menopausal by surgical oophorectomy as compared to those made climacteric by radiation therapy [35].

The Symptom Scale EORTC QLQ CX-24, revealed that there was a significant increase in “lymphoedema” and “peripheral neuropathy”. Lymphoedema may be attributed to lymph node damage by metastases. The significant increase in the peripheral neuropathy can be explained on the basis of post-radiation increase in the neurotoxicity [36].

The highly significant rise in the “sexual worry” item of the symptom scale is similar to the study, where the sexual worries were reported to be more than that of the control group. Our study analyzed the post-treatment quality of life which is noteworthy, as the time period chosen was 6 months post-treatment. This period is of great significance, as it is the time when the women cope up with the difficulties arising from the treatment and take over their responsibilities [37]. In the Indian context, it implies almost all the household chores and for most of the women even the financial responsibilities. Apart from these factors, the women who have recovered, fear recurrence.

### Development of Prediction Model

Based on these findings, we developed a prediction model tool for estimating the post treatment HRQoL outcome on Functional, Symptom and Global Health/QoL scales. During the development of the model, we compared the well established four machine learning algorithms, among which SVM(Linear) proved to be most accurate and consistent. To the best of our knowledge, this study is the first one to develop a prediction model for the post-treatment QoL. It also depicts that, given the same clinical and socio-demographic inputs; and output target classes, the predictive accuracy of SVM(Linear) is the highest and most consistent for HRQoL scales.

Recently, SVM and ANN models have been used for non-linear modelling in diverse fields, ranging from bioinformatics [38]–[40], neurosciences [41], [42], imaging [43], [44] to clinical prediction model development [45], [46].

We implemented univariate linear regression analysis during feature selection stage and validated our features by logistic regression coefficients values. Logistic regression was prioritized over linear regression due to multi-class nature of the classification. Cross validation with stratified K-Fold method was implemented to rule out varying percent of target class in a test set. This practice prevented biased predictions, over-fitting and hence provided with the accurate cross validation scores. We utilized AMI rather than Normalized Mutual Information index (NMI), as NMI fails to account for chance in comparing clustering performances by supervised learning algorithms [20] (See Appendix S1). The prediction model aims to determine the quality of life in an Indian context, which is essentially different from the developed countries in terms of socio-sexual environment and financial context which have been important determinants, as found in the study.

### Online Tool

We developed an online tool PrediQt-Cx, utilizing our prediction model, which is available at http://prediqt.org. The web application was developed using Python modules and back-end implementation of SVM (Linear) algorithm. The aim of developing the web application was to facilitate the accessibility of prediction model for critical evaluation, utilization for supporting treatment decisions and for external validation.

### Limitations

Our study was limited to north Indian population only. Thus, the applicability of model will be limited until external validation in cross-cultural and diverse socioeconomic settings is undertaken. Despite the multiple options being provided as treatment modality, the training of models and predictions were based only on three alternative sets of models (surgical treatment modalities, radio-chemotherapy and multiple interventions). This was decided as the sample sizes for some treatment modalities (conization, total abdominal hysterectomy and chemotherapy) were small and model training on them was not possible without prediction bias. Similarly FIGO (International Federation of Gynaecology and Obstetrics) stages were clubbed together into four groups. Grouping was based on stage specific clinical treatment decisions to account for small sample sizes in some stages (Stage IA1 and Stage IA2).

### Conclusion

In this study, the prediction model PrediQt-Cx, which was based on SVM, was developed and internally cross validated. After external validation, PrediQt-Cx can be employed in decision making procedure by clinicians and patients from north India region. It has been made available for open access at http://prediqt.org.

## Supporting Information

Appendix S1 **Model development and Adjusted Mutual Information (AMI).**
(PDF)
