# Supplementary material for: PrediQt-Cx: Post Treatment Health Related Quality of Life Prediction Model for Cervical Cancer Patients
Source: PLoS One. 2014 Feb 26;9(2):e89851. doi: 10.1371/journal.pone.0089851 (PMC3935936; doi:10.1371/journal.pone.0089851)
Supplement: Appendix S1 — Model development and Adjusted Mutual Information (AMI). (PDF) [file pone.0089851.s001.pdf]

Appendix S1

Section A

Model Development

Logistic Regression Model

Regularized logistic regression was applied to fit the data, as regularization helps in containing the over fitting or high variance problem [1]. We implemented logistic regression parameters θ using gradient descent as convex optimization algorithm with an objective to minimize the cost function. The regularized cost function $J(\theta)$ for our developed multi-class One-vs-All classifier can be represented as:

$$J(\theta) = \left[\frac{1}{m}\sum_{i=1}^{m}\left[-y^{(i)}log(h_\theta(x^{(i)})) - (1-y^{(i)})log(1-h_\theta(x^{(i)}))\right]\right] + \frac{\lambda}{2m}\sum_{j=1}^{n}\theta_j^2$$

where $h_\theta(x)$ is hypothesis given by the sigmoid function, $m, x^{(i)}$ and $y^{(i)}$represent training sample size, input and training class target respectively. Parameter θ_0 was not regularized while training the learning model as it was added as an intercept term and value set to 1. The gradient of the cost function is a vector where the jth element is defined as follows:

$$\frac{\partial J(\theta)}{\partial \theta_0} = \frac{1}{m}\sum_{i=1}^{m}\left(h_\theta(x^{(i)}) - y^{(i)}\right)x_j^{(i)} \qquad \text{for j} = 0$$

$$\frac{\partial J(\theta)}{\partial \theta_j} = \left(\frac{1}{m}\sum_{i=1}^{m}\left(h_\theta(x^{(i)}) - y^{(i)}\right)x_j^{(i)}\right) + \frac{\lambda}{m}\theta_j \qquad \text{for j} \geq 1$$

SVM Model

Support vector machines(SVMs) are a set of supervised learning methods which employ a subset of training points in the decision function (called support vectors) to construct a hyperplane or a set of hyperplanes in a high or infinite dimensional space. SVMs are versatile in decision function as different kernels can be specified for the same. A desirable separation is achieved by the hyperplane that has the largest functional margin, higher the margin the lower the generalization error of the classifier [2]. We

utilized Linear and Radial Basis function(RBF) kernels for model comparison.

Neural Network Model

The Artificial Neural Networks (ANN) model used in this study was a standard feed-forward, back-propagation neural network with three layers: an input layer, a hidden layer and an output layer. Our model consists of 13 input units, 10 hidden units and 1 output unit. The hidden neuron layer extracts important features contained in the input data. The cost function and the gradient of the implemented neural network with regularization is given by:

$$J(\theta) = \frac{1}{m}\sum_{i=1}^{m}\sum_{k=1}^{K}\left[-y_k^{(i)}log(h_\theta(x^{(i)}))_k - (1-y_k^{(i)})log(1-(h_\theta(x^{(i)}))_k)\right] + \frac{\lambda}{2m}\left[\sum_{j=1}^{20}\sum_{k=1}^{13}(\theta_{j,k}^{(1)})^2 + \sum_{j=1}^{1}\sum_{k=1}^{13}(\theta_{j,k}^{(2)})^2\right]$$

where $h_\theta(x)$ is hypothesis, m, x and y represents the sample size, training input and training target.

Section B

Adjusted Mutual Information (AMI)

Adjusted Mutual Information (AMI) accounts for the fact that the MI is generally higher for two clusterings with a larger number of clusters, regardless of whether there is actually more information shared. For two clusterings U and V, the AMI is given as:

$$AMI(U,V) = \frac{[MI(U,V) - E(MI(U,V))]}{[\max(H(U),H(V)) - E(MI(U,V))]}$$

This metric is independent of the absolute values of the labels, score values are independent of the permutation of the class or cluster label values.

References

1. Lee SI, Lee H, Abbeel P, Ng AY Efficient l1 regularized logistic regression. In: Proceedings of the Twenty-First National Conference on Artificial Intelligence (AAAI-06) (2006).

2. Guyon I, Weston J, Barnhill S, Vapnik V (2002) Gene selection for cancer classification using support vector machines. Machine learning 46: 389-422.
